# The role of perfusion, grey matter volume and behavioural phenotypes in the data-driven classification of cognitive syndromes

**DOI:** 10.1186/s13195-024-01410-1

**Published:** 2024-02-17

**Authors:** Ashwati Vipin, Bernett Teck Kwong Lee, Dilip Kumar, See Ann Soo, Yi Jin Leow, Smriti Ghildiyal, Faith Phemie Hui En Lee, Saima Hilal, Nagaendran Kandiah

**Affiliations:** 1 Dementia Research Centre (Singapore), 11 Mandalay Road, Lee Kong Chian School of Medicine, Nanyang Technology University, Singapore, 308232 Singapore; 2 Centre for Biomedical Informatics, 11 Mandalay Road, Lee Kong Chian School of Medicine, Nanyang Technological University, Singapore, 308232 Singapore; 3 Saw Swee Hock School of Public Health, Tahir Foundation Building, 12 Science Drive 2, National University of Singapore and National University Health System, Singapore, 117549 Singapore; 4 Duke-NUS Medical School, 8 College Road, Singapore, 169857 Singapore

**Keywords:** Cognitive syndromes, Data-driven classification, Neuroimaging, Grey matter perfusion, Grey matter volume, Behaviour

## Abstract

**Background:**

The use of structural and perfusion brain imaging in combination with behavioural information in the prediction of cognitive syndromes using a data-driven approach remains to be explored. Here, we thus examined the contribution of brain structural and perfusion imaging and behavioural features to the existing classification of cognitive syndromes using a data-driven approach.

**Methods:**

Study participants belonged to the community-based Biomarker and Cognition Cohort Study in Singapore who underwent neuropsychological assessments, structural-functional MRI and blood biomarkers. Participants had a diagnosis of cognitively normal (CN), subjective cognitive impairment (SCI), mild cognitive impairment (MCI) and dementia. Cross-sectional structural and cerebral perfusion imaging, behavioural scale data including mild behaviour impairment checklist, Pittsburgh Sleep Quality Index and Depression, Anxiety and Stress scale data were obtained.

**Results:**

Three hundred seventy-three participants (mean age 60.7 years; 56% female sex) with complete data were included. Principal component analyses demonstrated that no single modality was informative for the classification of cognitive syndromes. However, multivariate glmnet analyses revealed a specific combination of frontal perfusion and temporo-frontal grey matter volume were key protective factors while the severity of mild behaviour impairment interest sub-domain and poor sleep quality were key at-risk factors contributing to the classification of CN, SCI, MCI and dementia (*p* < 0.0001). Moreover, the glmnet model showed best classification accuracy in differentiating between CN and MCI cognitive syndromes (AUC = 0.704; sensitivity = 0.698; specificity = 0.637).

**Conclusions:**

Brain structure, perfusion and behavioural features are important in the classification of cognitive syndromes and should be incorporated by clinicians and researchers. These findings illustrate the value of using multimodal data when examining syndrome severity and provide new insights into how cerebral perfusion and behavioural impairment influence classification of cognitive syndromes.

## Background

There occurs significant heterogeneity in the presentation of pre-dementia and dementia stages with some individuals showing cognitive impairment, while others present with behavioural impairment. Both cognitive and behavioural phenotypes are related to changes in underlying brain structure and function including grey matter (GM) loss and perfusion deficits [1–3]. Since individuals typically progress from cognitively normal (CN), to subjective cognitive impairment (SCI), mild cognitive impairment (MCI) and dementia stages, it is important to integrate cognitive and biomarker data from CN, SCI, MCI and dementia to afford reliable classification of the individual along this spectrum. Currently, the classification of cognitive syndromes is largely carried out in clinical settings using neuropsychological assessments and structural MRI, where available. In this regard, understanding the clustering of multimodal factors in the classification of cognitive syndromes along the dementia spectrum is imperative for a more holistic view of which factors help distinguish between these syndromes. Additionally, detecting at-risk individuals, especially in asymptomatic early stages will allow for timely intervention and potentially delay progression along the dementia spectrum.

The contribution of MRI only or cognition only to the classification of subjects has also been assessed previously. Characterisation of heterogeneity in brain ageing, dementia and MCI have largely used data-driven clustering and neuroinformatic techniques [4–6]. These methods provide an unbiased method to classify syndromes along the dementia spectrum. Indeed, studies have shown how cortical thickness and grey matter volume (GMV) features by themselves or in combination with functional connectivity features can be helpful in the classification of dementia subtypes and MCI [6–9]. Prior findings have also illustrated separation between MCI and SCI using structural MRI [10]. Separately, studies have also examined comprehensive neuropsychological data and fluid biomarkers in phenotyping dementia subtypes [11, 12]. However, while some studies have assessed the combination of structural MRI and neuropsychological testing in subject categorisation, there exists a lack in multimodal subtyping studies [13]. There is thus a need for the development of multimodal fingerprints that combine data from different modalities including MRI, fluid biomarkers as well as neuropsychological performance to help improve the classification of dementia-related syndromes and sub-groups [14].

Some studies have examined the role of health-related behaviours such as diet, physical activity, smoking status and alcohol consumption on cognition and relative risk of dementia or cognitive performance [15, 16]. However, these behaviours tend to co-occur and thus their individual influence on cognition cannot be clearly defined. Similarly, some community-based studies have suggested that a combination of physical activity levels, smoking status and diet can allow for the identification of sub-groups at increased risk of dementia and may in turn benefit more from specific interventions [13]. Thus, there is merit in the assessment of combinations of factors in the classification of dementia-related syndromes. This approach will allow for the identification of groups of individuals that may benefit from specific interventions.

Findings do indicate that the use of multimodal imaging involving both structural as well as functional brain imaging is better at predicting dementia-related syndromes compared to single modality data alone [17]. In this regard, the use of structural and perfusion brain imaging in combination with behavioural information in the classification of cognitive syndromes remains to be explored. Moreover, employing data-driven clustering and multivariate feature selection methods using generalized linear models in a community-based cohort may provide important insights into the factors that contribute most to cognitive disorders in the community. This may in turn help assist identification of at-risk subgroups in a timely manner.

To address these gaps, we sought to examine the contribution of multimodal brain structural and perfusion imaging data and behavioural features to the classification of cognitive syndromes using a data-driven approach. We employed multivariate generalised linear models and aimed to evaluate which combination of features among brain structure, brain perfusion and behaviour contributed to the classification of CN, SCI, MCI and dementia syndromes.

## Methods

### Participants

Participants were recruited at the Dementia Research Centre (Singapore) as part of the ongoing Biomarker and Cognition Study. Three hundred seventy-three participants met the criteria for the current objective and were included. Inclusion criteria comprised the presence of a cognitive concern among individuals from the community aged between 30 and 95, inclusive of limits. A research diagnosis was assigned to each participant based on their cognitive performance. Participants were classified as cognitively normal if they had a CDR = 0, < 5 on the subjective memory complaints questionnaire and > 26 on the MoCA [18] Mean performance of CN individuals was calculated for the various cognitive domains listed earlier as part of the neuropsychological assessment. A participant with performance > 1.5 standard deviations below the CN mean on any cognitive domain and having no functional impairment was assigned as mild cognitive impairment, as per Petersen’s and National Institute on Aging-Alzheimer’s Association criteria [19, 20] Participants with CDR = 1 or more were assigned as dementia. Participants with subjective symptoms but not meeting the criteria for MCI and having no functional impairment were classified as SCI. Based on this criterion, out of the 373 subjects included in the study, 80 were classified as CN, 97 were classified as SCI, 192 were classified MCI and 4 met the criteria for dementia. Key exclusion criteria included illiteracy, diagnosis of major psychotic, psychiatric, neurological disorders and serious systemic disease.

### Neuropsychological and behavioural assessments

Trained research psychologists administered the following global cognitive tests: the Clinical Dementia Rating Scale [21], Montreal Cognitive Assessment [22], Visual Cognitive Assessment Test [23] and detailed neuropsychological test battery including tests for episodic memory [24–26], executive function [27, 28], language [22, 26], processing speed [28, 29] and visuospatial function [24, 28]. Participants were also administered behavioural questionnaires including the Mild Behaviour Impairment–Checklist(MBI-C) [30], Pittsburgh sleep quality index (PSQI) [31] and Depression Anxiety and Stress Scale(DASS) [32]. The MBI-C checklist domains of Interest, Mood, Control, Social and Beliefs were collected.

### Neuroimaging acquisition

All participants underwent whole-brain MRI scans using a 3T Siemens Prisma Fit scanner (Siemens Healthineers, Erlangen, Germany). The T1-weighted accelerated magnetization-prepared rapid gradient-echo sequence comprised the following parameters: repetition time = 2000 ms, echo time = 2.26 ms, inversion time = 800 ms, flip angle = 8°, matrix size = 256 × 256 and voxel size = 1.0 × 1.0 × 1.0 mm^3^. Additionally, 2D pulsed arterial spin labelling (ASL) data was also acquired from all participants. Scan parameters included: TR 2500 ms, T2 11 ms, TI = 1800 ms, bolus duration = 700 ms, flow limit = 100.0 cm/sec, field of view 256 mm, gap = 20.9 mm, distance factor 25%, flip angle 90°, 91 measurements (1 calibration M0, 45 label, 45 control) with voxel size 4.0 × 4.0 × 8.00 mm, matrix size = 64 × 64.

### Neuroimaging pre-processing and derivation of features

We used the Computational Anatomy Toolbox (http://dbm.neuro.uni-jena.de/cat12/) in Statistical Parametric Mapping (http://www.fil.ion.ucl.ac.uk/spm/), to process the T1 images for derivation of regional cortical GMV using the Automated Anatomical Labelling atlas [33]. All 3D T1-weighted MRI scans were normalised using an affine transformation followed by non-linear registration and corrected for bias field inhomogeneities. Images were then segmented to derive participant-level grey matter (GM), white matter, and cerebrospinal fluid components [33] The Diffeomorphic Anatomic Registration Through Exponentiated Lie algebra algorithm normalised the segmented scans into the standard MNI space to provide better precision in spatial normalisation to the template [34] Subsequently, the modulation step performed a non-linear deformation on the normalised segmented images which provides a comparison of the absolute amounts of tissue following correction for individual differences in brain size. All obtained segmented, modulated, and normalised grey matter images were then smoothed using an 8-mm full-width-half-maximum isotropic Gaussian smoothing kernel and the region-level grey matter volumes were derived using Computational Anatomy Toolbox functions covering the left and right cortical hemispheres of the brain.

ASL post-processing was performed using FSL’s Bayesian Inference for ASL MRI (BASIL) toolbox [35]. The acquired ASL scans were motion corrected using FSL’s MCFLIRT and calibrated based on the first unlabelled volume on the ASL scan. Spatial regularisation was applied prior to cerebral blood flow (CBF) calculation. CBF was quantified using the Buxton ASL kinetic model based on recommendations in the ASL white paper [35–37]. The generated CBF images were corrected for partial volume effects using BASIL’s adaptive spatial prior approach [38]. Here, T1-weighted images were registered to the ASL calibration scan using FSL’s FLIRT. The same transformation was applied to register the high-resolution partial volume maps to the ASL resolution. Partial volume corrected GM perfusion maps and GM CBF mean values were recorded for voxels with GM > 10%. For the derivation of the regional grey matter perfusion values, the GM segmentation maps were thresholded at 80% and the Harvard-Oxford cortical and subcortical atlas was applied to derive perfusion values in grey matter regions of interest. For this, standard space regions were transformed to native ASL space and voxels with a probability fraction > 0.5 were considered to lie within a region. At least 10 voxels must be found for perfusion values to be quantified in regions encompassing the GM cortical structures in ml/100 g/min.

### Statistical analysis

#### Features

Behavioural data features comprised total MBI-C score and five domains of Interest, Mood, Control, Social and Beliefs as well as DASS components of Depression, Anxiety, Stress and scores on the PSQI scale. MRI brain features comprised left and right cortical regions to maximise overlap between GMV and perfusion regions. Hippocampal subcortical areas were included in view of their specific contribution to cognitive processes. 

GMV regions included the left/right precentral gyrus, left/right superior frontal gyrus, left/right middle frontal gyrus, left/right inferior orbitofrontal, left/right medial superior frontal gyrus, left/right medial orbitofrontal, left/right insula, left/right middle cingulate cortex, left/right posterior cortex, left/right hippocampus, left/right parahippocampal gyrus, left/right lingual gyrus, left/right superior occipital gyrus, left/right inferior occipital gyrus, left/right fusiform gyrus, left/right postcentral gyrus, left/right superior parietal gyrus, left/right supramarginal gyrus, left/right angular gyrus, left/right precuneus, left/right superior temporal gyrus, left/right superior temporal pole, left/right middle temporal gyrus, left/right middle temporal pole.

ASL GM perfusion regions included left/right hippocampus, precuneus, posterior cingulate cortex, medial temporal, angular gyrus, insula, temporal pole, posterior superior temporal pole, posterior middle temporal, temporo-occipital middle temporal, superior parietal, anterior supramarginal, posterior supramarginal, superior lateral occipital, inferior lateral occipital, paracingulate, lingual, posterior temporal, temporo-occipital fusiform, occipital fusiform, occipital pole, frontal pole, superior frontal, middle frontal, precentral gyrus, postcentral gyrus, medial frontal, orbitofrontal, anterior cingulate, subcallosal.

Since neuropsychological test scores were utilised to establish participant research diagnosis, all neuropsychological test scores were excluded from the set of predictors to avoid circularity.

For overall sample characteristic comparisons, group comparisons for continuous variables were carried out using ANOVA with Tukey’s post hoc test. Group comparisons for categorical variables were carried out using chi-squared tests.

Principal component analysis (PCA) was performed for each of the categories of parameters to gain an understanding of the contribution of the parameters towards predicting cognitive syndromes as well as a general quality control check. Parameter readings used for the PCA were standardised and missing values assigned 0 which is the average value.

Kruskal-Wallis tests followed by Dunn’s post hoc testing were done to identify parameters which were associated with the cognitive syndromes of CN, SCI, MCI and dementia.

Glmnet was used to generate a multivariate linear regression model predictive of cognitive syndromes. The cognitive syndromes were assigned a value of 0 (CN), 1 (SCI), 2 (MCI) and 3 (dementia) for the purposes of the regression as an indicator of the severity of the condition. Parameters which are numeric in nature with a missing percentage less than 20% were used. The data was standardised prior to use and missing values assigned 0 which is the average value. 10-fold cross-validation was done for model selection and the final model presented. Model performance was assessed using a Kruskal-Wallis test of the model scores against the cognitive syndromes to determine whether the model scores are significantly different between cognitive syndromes. Pairwise comparisons of the model scores for the cognitive syndromes were also done using the receiver operator characteristics (ROC) curve. The thresholds for the ROC were determined as the best optimal combination of sensitivity and specificity and the area under the curve (AUC) reported.

All statistical analyses were conducted using R 4.2.2 with RStudio (2022.07.2). Statistical significance was deemed when the *P* values were less than 0.05. Multiple testing correction was performed using the method of Benjamini and Hochberg.

## Results

The analytic sample comprised 373 participants with cognitive, behavioural and neuroimaging data and categorised as CN, SCI, MCI and dementia. Overall, the groups differed in their age at visit, sex, education years and global cognition scores (Table 1) with the dementia group being the oldest and with the least education years.

**Table 1:** Participant demographics

	Cognitively normal (*n* = 80)	Subjective cognitive impairment (*n* = 97)	Mild cognitive impairment (*n* = 192)	Dementia (*n* = 4)	*p*-value
Age at visit, mean (SD)	56.9 (11.2)	58.1 (9.2)	63.3 (9.6)^a,b^	77.5 (11.2)^a,b,c^	< 0.001
Sex, female (*n*, %)	51 (63.8)	64 (65.9)	91 (47.4)	2 (50)	0.01
Education years, mean (SD)	14.5 (3.1)	14.6 (2.7)	13.6 (3.8)	8.5 (5.9)^a,b,c^	< 0.001
Visual Cognitive Assessment Test, mean (SD)	27.02 (2.3)	26.9 (2.3)	25.3 (3.7)^a,b^	17.5 (6.1)^a,b,c^	< 0.001
History of diabetes (*n*, %)	9 (11.3)	9 (9.3)	36 (18.8)	0 (0)	0.09
History of hypertension (*n*, %)	15 (18.8)	22 (22.7)	55 (28.6)	2 (50)	0.19
History of hyperlipidemia (*n*, %)	25 (31.3)	34 (35.1)	91 (47.6)	1 (25)	0.03

Superscript letters indicate whether group mean was significantly different compared with ^a^CN, ^b^SCI and ^c^MCI based on post-hoc comparisons (*p*<0.05) following one-way analysis of variance

Abbreviations: *CN* Cognitively normal, *SCI* Subjective cognitive impairment, *MCI* Mild cognitive impairment

### Principal component analyses across regional grey matter volume, regional grey matter perfusion and behavioural data

**Fig. 1:**
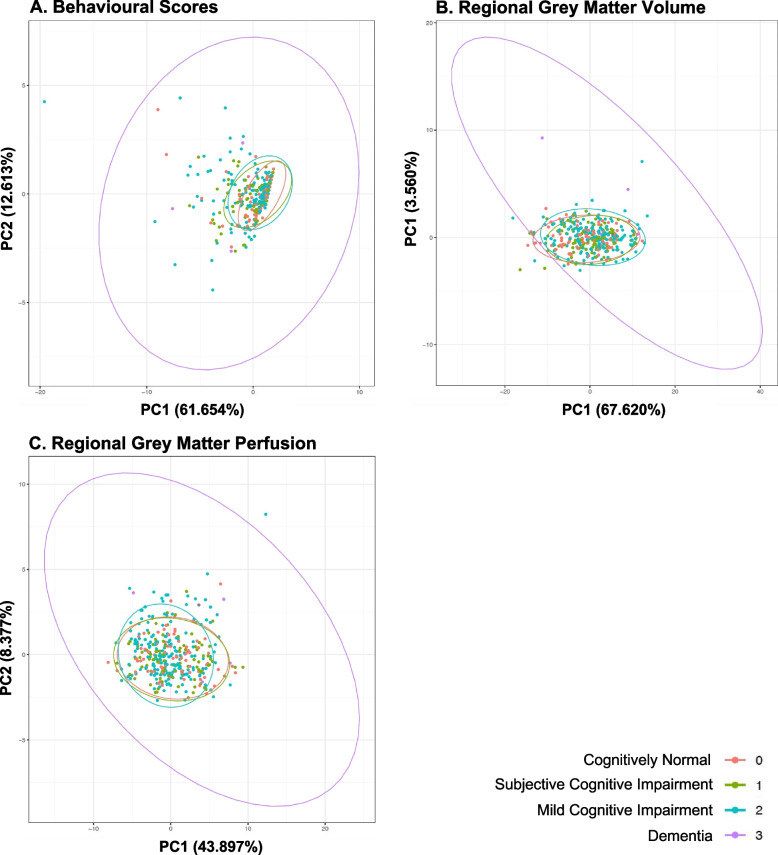
Principal component analyses for behavioural scores (**A**), regional grey matter volumes (**B**) and regional grey matter perfusion (**C**). Clustering of data across behavioural, grey matter volumes and grey matter perfusion did not differ across cognitive syndromes

Principal component analysis was performed for behavioural data, regional GMV and regional perfusion. Dimensionality reduction in all data types did not reveal difference in data clustering across the different cognitive syndromes, suggesting that no principal components within each data type distinguished between cognitive syndromes (Fig. 1A–C).

We carried out Kruskal-Wallis analysis and Dunn’s post-hoc tests to understand which parameters were able to differentiate between cognitive syndromes. Significant results are reported at FDR-corrected *p* < 0.10 threshold in Table 2. Specifically, a few parameters representing behaviour measures, GMV and GM perfusion in cortical regions of interest, provided preliminary indication that these features may be useful in the classification of cognitive syndromes which led to further Glmnet testing.

**Table 2:** Parameters illustrating differences between cognitively normal, subjective cognitive impairment, mild cognitive impairment and dementia stages

Parameter	Category	FDR adjusted *p*-value	*n*	Chi-sq
Age	Demographics	< 0.001	373	39.23
Left hippocampal volume	Grey matter volume	0.02	373	17.98
Right hippocampal volume	Grey matter volume	0.07	373	13.91
Left middle cingulate cortex	Grey matter volume	0.07	373	13.76
MBI-C control	Behavioural survey	0.09	372	12.01
Precentral gyrus	Grey matter perfusion	0.07	371	13.40
Middle frontal gyrus	Grey matter perfusion	0.09	314	11.98

Abbreviations: *MBI-C* Mild Behaviour Impairment - Checklist

### Multivariate generalized linear regression

**Fig. 2:**
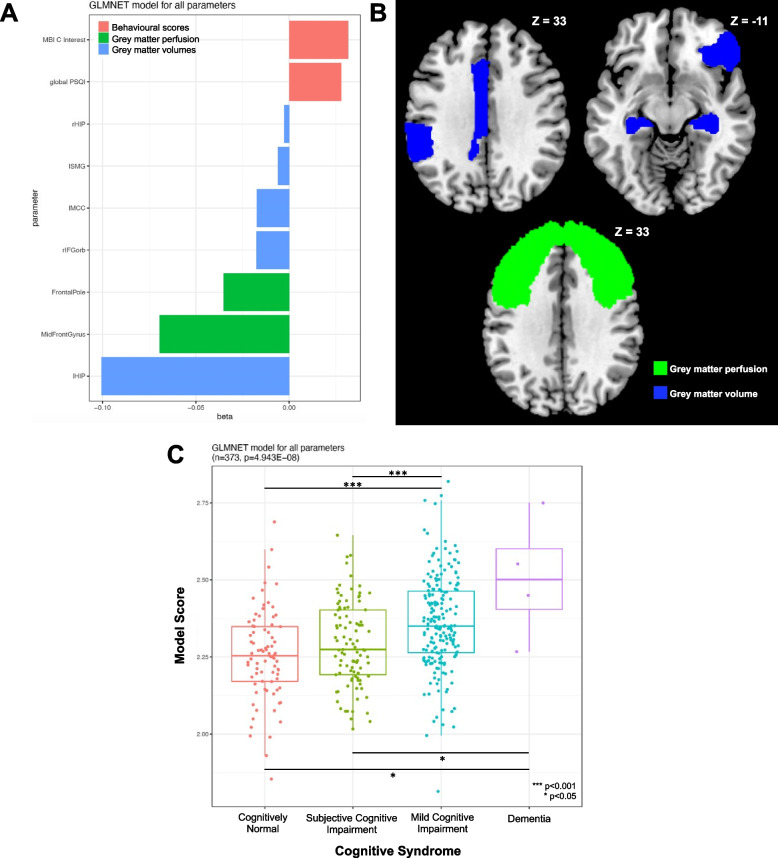
Glmnet analyses revealed a combination of regional grey matter volume, perfusion and behavioural scores as discriminative between cognitive syndromes. **A** Higher temporal, frontal and parietal grey matter volume and higher frontal perfusion were protective against worsening cognitive syndrome. Higher scores on the PSQI (worse sleep quality) and MBI-C Interest (worse behaviour) were associated with increased risk of more advanced cognitive syndromes. **B** Brain grey matter regions where increased grey matter volume and perfusion were protective against worsening cognitive syndrome. **C** Differences in glmnet model scores revealed significant differences between groups in a step-wise manner. Abbreviations: MBI-C, mild behaviour impairment checkline; PSQI, Pittsburgh sleep quality index; rHIP, right hippocampus; lSMG, left supramarginal gyrus; lMCC, left mid cingulate cortex; rIFGorb, right inferior fronto-orbital gyrus; lHIP, left hippocampus

We used glmnet to identify a set of parameters for the classification of cognitive syndromes and differentiate between CN, SCI, MCI and dementia (Fig. 2A–B). Higher left and right hippocampal volume, right inferior fronto-orbital gyrus, left middle cingulate cortex and left supramarginal gyrus GMV comprised key protective factors against worsening cognitive syndromes. Additionally, higher frontal pole and middle frontal gyrus perfusion comprised key protective factors against worsening cognitive syndromes. On the other hand, higher MBI-C interest domain scores and global PSQI score indicated worse behaviour and sleep quality, respectively and were risk factors for worsening cognitive syndromes.

Kruskal-Wallis tests indicated a large difference between model scores with a step-wise increase across CN, SCI, MCI and dementia groups (*p* < 0.001; Fig. 2C). Post hoc Dunn’s test indicated that pair-wise differences in model scores were especially prominent between CN vs MCI (*p* < 0.001), CN vs dementia (*p* = 0.01), SCI vs MCI (*p* < 0.001) and SCI vs dementia (*p* = 0.026). Model performance did not significantly differ between CN vs SCI and MCI vs dementia.

### Calculation of model prediction accuracy in distinguishing between cognitive syndromes

**Fig. 3:**
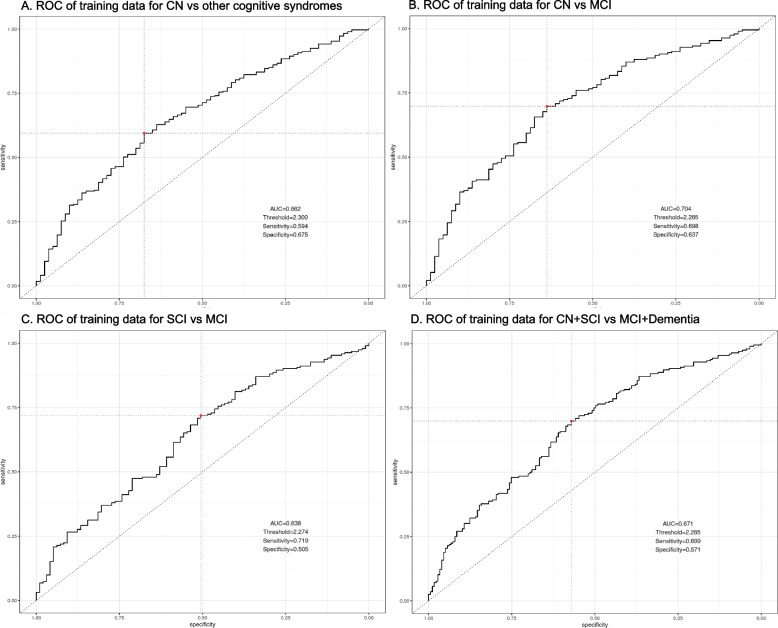
ROC curves of pairwise comparisons of model scores between cognitive syndromes. **A** Cognitively normal versus other cognitive syndromes, **B** Cognitively normal versus mild cognitive impairment, **C** Subjective cognitive impairment versus mild cognitive impairment, **D** grouping of cognitively normal + subjective cognitive impairment versus mild cognitive impairment + dementia. Abbreviations: ROC, receiver operator characteristics; CN, cognitively normal; SCI, subjective cognitive impairment; MCI, mild cognitive impairment

To assess model prediction accuracy, we ran a series of ROC analyses to identify the best threshold (best combination of sensitivity and specificity) for the model score and then used this threshold to compute the performance metrics. This analysis showed an AUC of 0.662 (sensitivity = 0.594; specificity = 0.675; Fig. 3A) for the classification of CN versus any other cognitive syndrome. For the classification of MCI vs CN, the ROC showed an AUC of 0.704 (sensitivity = 0.698; specificity = 0.637; Fig. 3B). For the classification of MCI vs SCI, the ROC showed an AUC of 0.638 (sensitivity = 0.719; specificity = 0.505; Fig. 3C). After grouping of CN + SCD and MCI + dementia and classification of either of these syndromes, the model showed an AUC of 0.671 (sensitivity = 0.699; specificity = 0.571; Fig. 3D).

## Discussion

This study examined the role of multimodal brain MRI and behavioural data in the classification of neurocognitive syndromes comprising CN, SCI, MCI and dementia. In separate PCA analyses, no single modality was informative for cognitive syndrome classification. However, multivariate glmnet analyses revealed that a specific combination of GM perfusion, GMV and behavioural impairment provided crucial information for the discrimination between cognitive syndromes. Specifically, frontal perfusion and temporo-frontal GMV were key protective factors while the severity of MBI-C Interest sub-domain and PSQI sleep quality were key at-risk factors contributing to CN, SCI, MCI and dementia stages in a step-wise manner. Moreover, based on these results, the glmnet model showed best classification accuracy in differentiating between CN and MCI. Our findings emphasise the need for multimodal information over unimodal data types in the understanding and classification of cognitive syndromes. The combination of biomarker data and cognitive-behavioural data may be advantageous to improve the accuracy of cognitive syndrome classification.

The use of multivariate glmnet in our study enabled us to examine all possible combinations of GMV, perfusion and behavioural measures. Employing this approach, we were able to obtain the combination of high-performing features to discriminate between cognitive syndromes. Additionally, the combination of features picked by glmnet indicated a step-wise increment in model score predictability in discriminating between CN, SCI, MCI and dementia. Such a multi-variate data-driven approach to assess the classification accuracy of cognitive syndromes enabled the identification of best-performing features, as observed in previous studies [39]. We found that GMV, perfusion and behavioural impairment variables carry useful information for the classification of cognitive syndromes with high levels of accuracy, sensitivity and specificity. Following further validation, this set of features can potentially be included in clinical settings to gain insights into cognitive syndrome severity and staging.

Our study provides novel insights into the role of GM perfusion as well as behavioural impairment in the classification of cognitive syndromes, in addition to previously reported GMV measures [4]. Indeed, population-based studies have examined cerebral perfusion data, cross-sectionally and longitudinally to assess dementia risk and cognitive decline [1]. These indicate that lower cerebral perfusion is associated with a higher risk of dementia, which is in line with our findings indicating higher cerebral blood flow in frontal GM is protective against more severe stages of cognitive impairment [1]. Additionally, lower baseline perfusion was associated with accelerated cognitive decline, especially in individuals with greater small vessel disease involving white matter hyperintensity burden [1]. We have also shown previously that frontal lobe white matter hyperintensity burden relates to widespread GM atrophy in MCI [40]. Our findings of higher frontal perfusion being protective against more severe cognitive syndromes are thus an important addition to the current understanding of the contribution of cerebral perfusion to cognitive impairment. Notably, prior studies point towards small vessel disease being an important factor in this relationship between cognitive performance and brain perfusion. However, how regional perfusion contributes to this relationship needs further comprehensive examination in future studies.

Our findings indicate that the combination of GMV, perfusion and behaviour was important not only in the classification of cognitive syndromes but also in the separation between SCI from MCI and dementia. In this regard, prior studies have utilised clustering approaches on structural MRI data to illustrate subtypes of SCI, involving no atrophy, diffuse atrophy and AD-like temporal atrophy and their respective cognitive decline [10]. Additionally, recent studies have also highlighted the presence of behavioural features involving depressive symptoms in subtypes of SCI [41]. Certain trajectories of SCI may also be predictive of further decline to MCI and dementia [42]. Furthermore, the presence of MBI is thought to represent underlying neurodegenerative disease [43]. Indeed, performance on the MBI-C, along with the presence of SCI, has also been shown to be associated with a greater risk of cognitive decline and progression to dementia [44]. The presence of sleep deficits has also been shown to be associated with behavioural impairment as well as more severe SCI [45, 46]. Thus, our findings of temporal dominant GMV as a key protective factor and behavioural impairment as an at-risk feature of more severe cognitive impairment, both between SCI and MCI as well as SCI and dementia, add to the existing body of literature illustrating distinct signatures in SCI. Notably, the selection of frontal cerebral brain perfusion features in distinguishing between SCI from MCI and dementia provides additional novel insights to the understanding of underlying differences between cognitive syndromes using a data-driven approach.

The strengths of this study include the use of comprehensive neuropsychological assessments for the establishment of research diagnosis. Additionally, participants are from a community-based cohort in Singapore, thus representing the larger proportion of MCI patients. Future directions will include validation of these features in larger samples to assess classification accuracy. Notably, prior studies have not combined the use of perfusion measures with GMV as well as behavioural performance in the classification of cognitive syndromes. This multimodal approach in our study has thus illustrated how unimodal approaches may not be comprehensive in examining differences between early stages of cognitive impairment. In this regard, the use of glmnet has provided an unbiased and comprehensive means to assess multivariate feature combinations that are best at differentiating between stages of cognitive impairment.

There are some limitations to this study. These include the small sample of subjects with dementia due to the community-based nature of the study cohort. We included this group of participants to test and illustrate the degree of distinction along the entire cognitive impairment spectrum. The lack of a biomarker-based classification of our patients into AD and non-AD is also a limitation. However, given that this is a community-based cohort, we feel that the clinical-based classification of MCI and dementia is more reflective of real-life practice. Future studies will focus on increasing the pool of dementia participants to further validate this set of features. Additionally, these results will need to be further validated in a longitudinal study to better assess the prediction accuracy of the selected features in distinguishing between cognitive syndromes. We did not assess the influence of vascular risk factors on the classification of cognitive syndromes and cognitive decline, which will be a key aspect of future studies. Additionally, the use of a pulsed ASL image sequence due to scanner limitations is a drawback in this study as it may involve a lower ASL signal-to-noise ratio compared to other ASL imaging sequences. Future studies will aim for the implementation of improved ASL sequences.

## Conclusions

In conclusion, this study attempts to bring together multiple data modalities to identify features that would best classify cognitive syndromes from a cross-sectional community-based cohort. The generalized linear model analyses identified fronto-temporal GMV and frontal GM perfusion as key protective factors against more severe stages of cognitive impairment. Concurrently, higher MBI-C interest domain and poorer sleep quality scores increased the risk of more severe cognitive impairment. This combination of features had the highest prediction accuracy in distinguishing between CN, SCI, MCI and dementia. These findings indicate the value of using multimodal data when examining syndrome severity and provide new insights into how cerebral perfusion measures and behavioural impairment can influence the classification of cognitive syndromes.

## Data Availability

Anonymized data will be shared by request from any qualified investigator.

## Abbreviations

CN: Cognitively normal
SCI: Subjective cognitive impairment
MCI: Mild cognitive impairment
GM: Grey matter
GMV: Grey matter volume
MBI-C: Mild behaviour impairment–checklist
PSQI: Pittsburgh sleep quality index
DASS: Depression anxiety and stress scale
BASIL: Bayesian inference for ASL MRI
PCA: Principal component analysis
ROC: Receiver operator characteristics
AUC: Area under the curve
ASL: Arterial spin labelling
CBF: Cerebral blood flow

## References

[1] Wolters FJ, Zonneveld HI, Hofman A, Van Der Lugt A, Koudstaal PJ, Vernooij MW (2017). Cerebral perfusion and the risk of dementia: a population-based study. Circulation..

[2] Karas GB, Scheltens P, Rombouts SARB, Visser PJ, Van Schijndel RA, Fox NC (2004). Global and local gray matter loss in mild cognitive impairment and Alzheimer’s disease. Neuroimage..

[3] Seeley WW, Crawford RK, Zhou J, Miller BL, Greicius MD (2009). Neurodegenerative diseases target large-scale human brain networks. Neuron [Internet]..

[4] Habes M, Grothe MJ, Tunc B, McMillan C, Wolk DA, Davatzikos C (2020). Disentangling heterogeneity in Alzheimer’s disease and related dementias using data-driven methods. Biol Psychiatry..

[5] Noh Y, Jeon S, Lee JM, Seo SW, Kim GH, Cho H (2014). Anatomical heterogeneity of Alzheimer disease: based on cortical thickness on MRIs. Neurology..

[6] Zhang X, Mormino EC, Sun N, Sperling RA, Sabuncu MR, Yeo BTT (2016). Bayesian model reveals latent atrophy factors with dissociable cognitive trajectories in Alzheimer’s disease. Proc Natl Acad Sci U S A..

[7] Habes M, Sotiras A, Erus G, Toledo JB, Janowitz D, Wolk DA (2018). White matter lesions: Spatial heterogeneity, links to risk factors, cognition, genetics, and atrophy. Neurology..

[8] Eavani H, Habes M, Satterthwaite TD, An Y, Hsieh MK, Honnorat N (2018). Heterogeneity of structural and functional imaging patterns of advanced brain aging revealed via machine learning methods. Neurobiol Aging..

[9] Kim HJ, Park JY, Seo SW, Jung YH, Kim Y, Jang H (2019). Cortical atrophy pattern-based subtyping predicts prognosis of amnestic MCI: an individual-level analysis. Neurobiol Aging..

[10] Jung NY, Seo SW, Yoo H, Yang JJ, Park S, Kim YJ (2016). Classifying anatomical subtypes of subjective memory impairment. Neurobiol Aging..

[11] Van Der Vlies AE, Verwey NA, Bouwman FH, Blankenstein MA, Klein M, Scheltens P (2009). CSF biomarkers in relationship to cognitive profiles in Alzheimer disease. Neurology..

[12] Martorelli M, Sudo FK, Charchat-Fichman H (2019). This is not only about memory: A systematic review on neuropsychological heterogeneity in Alzheimer’s disease. Psychol Neurosci..

[13] Sun N, Mormino EC, Chen J, Sabuncu MR, BTT Y. Multi-modal latent factor exploration of atrophy, cognitive and tau heterogeneity in Alzheimer’s disease. Neuroimage [Internet]. 2019;201 [cited 2023 Apr 12]. Available from: https://pubmed.ncbi.nlm.nih.gov/31344486/. 10.1016/j.neuroimage.2019.116043 31344486

[14] Rashid B, Calhoun V (2020). Towards a brain-based predictome of mental illness. Hum Brain Mapp [Internet]..

[15] Peters R, Booth A, Rockwood K, Peters J, D’Este C, Anstey KJ (2019). Combining modifiable risk factors and risk of dementia: a systematic review and meta-analysis. BMJ Open [Internet]..

[16] Dingle SE, Bowe SJ, Bujtor M, Milte CM, Daly RM, Anstey KJ (2022). Associations between data-driven lifestyle profiles and cognitive function in the AusDiab study. BMC Public Health [Internet]..

[17] Calhoun VD, Pearlson GD, Sui J (2021). Data-driven approaches to neuroimaging biomarkers for neurological and psychiatric disorders: emerging approaches and examples. Curr Opin Neurol [Internet]..

[18] Youn JC, Kim KW, Lee DY, Jhoo JH, Lee SB, Park JH (2009). Development of the Subjective Memory Complaints Questionnaire. Dement Geriatr Cogn Disord..

[19] Jack CR, Bennett DA, Blennow K, Carrillo MC, Dunn B, Haeberlein SB (2018). NIA-AA Research Framework: Toward a biological definition of Alzheimer’s disease. Alzheimer’s Dementia..

[20] Petersen RC, Caracciolo B, Brayne C, Gauthier S, Jelic V, Fratiglioni L (2014). Mild cognitive impairment: a concept in evolution. J Intern Med..

[21] Morris JC (1997). Clinical dementia rating: a reliable and valid diagnostic and staging measure for dementia of the Alzheimer type. Int Psychogeriatr..

[22] Ng A, Chew I, Narasimhalu K, Kandiah N (2013). Effectiveness of Montreal Cognitive Assessment for the diagnosis of mild cognitive impairment and mild Alzheimer’s disease in Singapore. Singapore Med J..

[23] Kandiah N, Zhang A, Bautista DC, Silva E, Ting SKS, Ng A (2016). Early detection of dementia in multilingual populations: Visual Cognitive Assessment Test (VCAT). J Neurol Neurosurg Psychiatry..

[24] Schmidt M (1996). Rey Auditory and Verbal Learning Test. A handbook.

[25] Rey A. L'examen psychologie dans les cas d'enceaphalopathie traumatique (Les problémes). Archives de Psychologie. 1941;28:286–340.

[26] Wechsler D. Wechsler Memory Scale--Fourth Edition (WMS-IV). APA PsycTests. 2009; 10.1037/t15175-000.

[27] Arbuthnott K, Frank J (2000). Trail Making Test, Part B as a Measure of Executive Control: Validation Using a Set-Switching Paradigm. J Clin Exp Neuropsychol..

[28] Wechsler D. Wechsler Adult Intelligence Scale--Fourth Edition (WAIS-IV) [Internet]. APA PsycTests. 2008; 10.1037/t15169-000.

[29] D’Elia LF, Satz P, Uchiyama C, White T (1994). Colour Trails 1 (CT1).

[30] Ismail Z, Agüera-Ortiz L, Brodaty H, Cieslak A, Cummings J, Fischer CE (2017). The Mild Behavioral Impairment Checklist (MBI-C): A rating scale for neuropsychiatric symptoms in pre-dementia populations HHS Public Access. J Alzheimers Dis..

[31] Buysse DJ, Reynolds CF, Monk TH, Berman SR, Kupfer DJ (1989). The Pittsburgh Sleep Quality Index: a new instrument for psychiatric practice and research. Psychiatry Res..

[32] Lovibond S, Lovibond P (1995). Manual for the depression anxiety and stress scales (DASS21).

[33] Ashburner J (2007). A fast diffeomorphic image registration algorithm. Neuroimage..

[34] Ashburner J, Friston KJ (2005). Unified segmentation. Neuroimage..

[35] Chappell MA, Groves AR, Whitcher B, Woolrich MW (2009). Variational Bayesian inference for a nonlinear forward model. IEEE Trans Signal Process..

[36] Alsop DC, Detre JA, Golay X, Günther M, Hendrikse J, Hernandez-Garcia L (2015). Recommended implementation of arterial spin-labeled perfusion MRI for clinical applications: A consensus of the ISMRM perfusion study group and the European consortium for ASL in dementia. Magn Reson Med..

[37] Buxton RB, Frank LR, Wong EC, Siewert B, Warach S, Edelman RR (1998). A general kinetic model for quantitative perfusion imaging with arterial spin labeling. Magn Reson Med..

[38] Chappell MA, Groves AR, MacIntosh BJ, Donahue MJ, Jezzard P, Woolrich MW (2011). Partial volume correction of multiple inversion time arterial spin labeling MRI data. Magn Reson Med..

[39] Casanova R, Whitlow CT, Wagner B, Williamson J, Shumaker SA, Maldjian JA (2011). High dimensional classification of structural MRI Alzheimer’s disease data based on large scale regularization. Front Neuroinform..

[40] Vipin A, Foo HJL, Lim JKW, Chander RJ, Yong TT, Ng ASL (2018). Regional white matter hyperintensity influences grey matter atrophy in mild cognitive impairment. J Alzheimer’s Dis..

[41] Ribaldi F, Rolandi E, Vaccaro R, Colombo M, Battista Frisoni G, Guaita A. The clinical heterogeneity of subjective cognitive decline: a data-driven approach on a population-based sample. Age Ageing. 2022;51(10). 10.1093/ageing/afac209 36273347

[42] Liew TM. Trajectories of subjective cognitive decline, and the risk of mild cognitive impairment and dementia. Alzheimers Res Ther. 2020;12(1). 10.1186/s13195-020-00699-y PMC7592368 33109275

[43] Creese B, Ismail Z (2022). Mild behavioral impairment: measurement and clinical correlates of a novel marker of preclinical Alzheimer’s disease. Alzheimers Res Ther..

[44] Ismail Z, McGirr A, Gill S, Hu S, Forkert ND, Smith EE (2021). Mild Behavioral Impairment and Subjective Cognitive Decline Predict Cognitive and Functional Decline. J Alzheimers Dis..

[45] Exalto LG, Hendriksen HMA, Barkhof F, van den Bosch KA, Ebenau JL, van Leeuwenstijn-Koopman M, et al. Subjective cognitive decline and self-reported sleep problems: The SCIENCe project. Alzheimers Dement (Amst). 2022;14(1). 10.1002/dad2.12287 PMC9107682 35603141

[46] Joo HJ, Joo JH, Kwon J, Jang BN, Park EC (2021). Association between quality and duration of sleep and subjective cognitive decline: a cross-sectional study in South Korea. Sci Rep..

